# Long Noncoding RNAs and Stress Response in the Nucleolus

**DOI:** 10.3390/cells8070668

**Published:** 2019-07-02

**Authors:** Sergei A. Pirogov, Vladimir A. Gvozdev, Mikhail S. Klenov

**Affiliations:** Institute of Molecular Genetics, Russian Academy of Sciences, 2 Kurchatov Sq., 123182 Moscow, Russia

**Keywords:** lncRNA, nucleolus, ribosomal RNA, stress, pRNA, intergenic spacers, noncoding RNA, chromatin, PAPAS, SLERT

## Abstract

Long noncoding RNAs (lncRNAs) perform diverse functions in the regulation of cellular processes. Here we consider a variety of lncRNAs found in the ribosome production center, the nucleolus, and focus on their role in the response to environmental stressors. Nucleolar lncRNAs ensure stress adaptation by cessation of resource-intensive ribosomal RNA (rRNA) synthesis and by inducing the massive sequestration of proteins within the nucleolus. Different cell states like quiescence and cancer are also controlled by specific lncRNAs in the nucleolus. Taken together, recent findings allow us to consider lncRNAs as multifunctional regulators of nucleolar activities, which are responsive to various physiological conditions.

## 1. Introduction

Noncoding transcripts longer than 200 nucleotides, known as long noncoding RNAs (lncRNAs) act as specific regulators of gene expression, chromosomal dynamics, RNA modifications and as architectural components assembling protein complexes [1,2]. In this review, we discuss current data related to the functions of lncRNAs in the nucleolus, the most prominent nuclear membrane-less compartment. The nucleolus serves as a factory for the assembly of ribosomal particles gathering all needed participants for rRNA maturation and plays an important role in the sequestration of many nucleoplasmic proteins, especially those that are important for cell cycle progression and stress adaptation [3]. The multitasking roles of the nucleolus in development, tumorigenesis, DNA repair and cell cycle progression have been considered in many recent reviews [4,5,6,7]. Here, we focus on the molecular details of the nucleolar response to abnormal and stressful conditions, carried out with the participation of lncRNAs. Studying the mechanisms of cellular reactions to stress is also important for understanding pathogenic processes in various diseases. For example, stresses like heat and osmotic shocks lead to protein misfolding, which is common with type II diabetes, Alzheimer, Huntington and Parkinson diseases, while oxidative stress may reflect the course of cardiovascular diseases and cancer [8,9]. Cells coping with stress undergo a deep reorganization of gene regulation and trigger various stress-induced reactions, including activation of chaperones, proteasomal degradation of misfolded proteins and repression of rRNA synthesis, as this cellular activity requires tremendously high energy consumption [10,11]. The functions of lncRNAs in stress responses include the regulation of translation, apoptosis, cell cycle progression, as well as the modulation of nucleolar activities [12,13,14]. 

A fundamentally new stage in cell biology is represented by the emergence of a paradigm, which considers some cellular compartments, including the nucleolus, as biomolecular condensates that are organized by liquid-liquid phase separation (LLPS) and exhibit high sensitivity to environmental cues [15,16,17]. Formation of membrane-less bodies through LLPS is thought to be based on three pillars: multidomain proteins, intrinsically disordered proteins that lack a fixed three-dimensional structure and an RNA component [17,18,19,20,21,22,23]. Nucleolus assembly depends on ribosomal DNA (rDNA) transcription, since pre-rRNAs act as seed molecules recruiting abundant nucleolar proteins, such as nucleolin, fibrillarin and nucleophosmin. These proteins contain disordered low-complexity regions, which are required for effective LLPS [24,25]. Interestingly, the nucleolar integrity is also provided by non-coding RNAs originating from intronic Alu elements (aluRNAs) [26,27]. A sophisticated inner structure of amniotic nucleoli includes the Fibrillar Center (FC), the Dense Fibrillar Components (DFC) and the Granular Component (GC). rDNA transcription occurs at the interface between the FC and DFC, processing of pre-rRNA takes place mainly in the DFC, while pre-ribosomal subunits are finally assembled and stored in the GC. Impressive in vivo and in vitro experiments demonstrated the multiphase droplet nature of nucleoli and revealed that DFC and GC behave like immiscible liquid phases [25]. 

## 2. Epigenetic Regulation of rRNA Synthesis 

**Figure 1:**
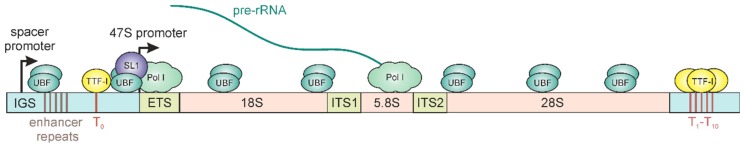
Scheme of a mouse rRNA gene with associated proteins. The pre-rRNA coding region consists of 18S, 5.8S and 28S rRNA sequences and transcribed ETS and ITS spacers. Individual rDNA repeats are separated by IGSs. Pol I transcription at the 47S promoter is initiated by specific set of proteins, which includes UBF and SL1 complex. The dimers of UBF are also associated with the pre-rRNA coding regions of active rDNA repeats. Transcription ends at multiple terminator elements (T_1–10_) that bind TTF-I protein. In addition, TTF1 occupies a terminator-like sequence (T_0_) near the rDNA promoter. IGSs also contain repetitive enhancer elements and spacer promoters, which in mice are located about 2 kb upstream of the pre-rRNA transcription start site.

The nucleolus is built around rDNA clusters that include hundreds of tandemly repeated rRNA genes separated by intergenic spacer regions (IGS). Chromosome parts that contain such clusters are termed NORs (Nucleolar Organizing Regions), which in human cells are located on the short arms of the five acrocentric chromosomes [28]. The IGS regions vary in length from 2 to 30 kb in eukaryotes, whereas the pre-rRNA coding regions have a relatively similar length, ranging from 7 to 13 kb. pre-rRNA transcripts (47S pre-rRNA in mammals) consist of 18S, 5.8S and 28S rRNA sequences and external and internal transcribed spacers (ETS, ITS), which are eliminated during processing (Figure 1). Transcription starts from an rDNA promoter that includes a core promoter and UCE (upstream control element). In addition, the presence of intergenic transcript promoters was demonstrated in IGSs of different animals, including vertebrates (*Xenopus laevi*, *Mus musculus* and *Homo sapiens*) and invertebrates (*Drosophila melanogaster*) [29,30,31]. IGS regions also contain repetitive enhancer sequences, as well as terminator elements (T), which are found both upstream (T_0_) and downstream (T_1–10_) of the pre-rRNA coding region. 

The level of rRNA synthesis is extremely flexible. It depends on different environmental conditions, as well as the stage of the cell cycle, and is tightly regulated by various metabolic and signaling pathways [14,32,33,34]. Although rRNA genes are the most actively transcribed genes in eukaryotes, heterochromatin plays an essential role in the maintenance of rDNA stability preventing recombination between rDNA repeats and ensuring nucleolar structure [35,36]. Importantly, only a portion of rRNA genes in eukaryotic genomes are active, while the others are usually reversibly inactivated [37,38]. In addition to the providing genome stability, this regulation is thought to have an adaptive role in preserving energy resources that could have been wasted on excessive rRNA transcription. While some individual NORs can be completely repressed [39], even inside active NORs only a fraction of the rDNA repeats seem to be transcribed [40]. Some of the inactivated rRNA genes are represented by damaged copies, an effect that creates an additional level of regulatory complexity. For instance, approximately 20–30% of human rDNA repeats form noncanonical and palindromic structures [40,41]. Another example of the coexistence of active and inactive rDNA units within the same NOR comes from the studies of rDNA loci in insects. Up to 40% of *Drosophila* rRNA genes carry insertions of the specific retrotransposons R1 and R2 in the 28S rRNA sequence. Although inserted rDNA repeats may contain normal promoters, they are transcribed several orders of magnitude lower compared to non-inserted rRNA genes [42]. Generally, the mechanisms of repression of individual rDNA units remain obscure. 

Transcription of rDNA is performed by RNA polymerase I (Pol I) and requires a unique set of basal factors. In mammals, rDNA transcription initiation depends on the binding of the dimeric UBF protein (upstream binding factor) that recruits the SL1 complex (TBP and TAFs) and the initiation factor TIF-IA (RRN3) to the rDNA promoter (Figure 1) [43,44,45,46]. The UBF was also shown to be associated along the entire length of active rRNA gene body [47,48]. Transcription ends at multiple terminator elements that bind TTF-I protein, which also occupies the upstream element T_0_ (Figure 1) and serves as an important transcription factor recruiting both activators and repressors of rRNA synthesis [49,50,51]. The promoter and terminator regions of active rRNA genes are in close proximity, forming a loop from the interaction between TTF1 molecules and other factors and allowing efficient re-initiation of Pol I transcription [52,53,54].

In terms of epigenetic marks, promoters of transcriptionally active rRNA genes in mammalian cells are usually characterized by hypomethylated DNA at CpG dinucleotides, acetylation of histone H4, and dimethylation of histone H3 at lysine 4 (H3K4me2). Numerous studies also suggest that the bodies of active rRNA genes contain few nucleosomes or may even be completely free of histones [46,48], an effect that is largely discussed in recent reviews [43,55]. On the contrary, silent rRNA genes can be marked by DNA hypermethylation and regular nucleosomes carrying repressive histone marks H3K9me3, H4K20me3 and H3K27me3, as well as deacetylated H4 histones (see [43,55,56,57] for reviews of rDNA chromatin organization). However, in some cases, transcriptionally inactive rDNA repeats were shown to be non-methylated at CpG sites or non-nucleosomal [51,58]. 

In addition, promoters of active and silent rDNA repeats contain differently positioned nucleosomes. At active genes, the promoter-bound nucleosome covers nucleotides from −157 to −2 upstream of the transcription start site, facilitating cooperative binding of transcription initiation factors. At silent rRNA genes, the nucleosome is positioned 24 nt downstream, preventing transcription initiation [59].

Although a substantial part of rDNA repeats normally undergo selective heterochromatinization and inactivation, stressful conditions can lead to repression of a much larger number of rRNA genes, or even complete cessation of rDNA transcription. First of all, down-regulation of rRNA synthesis is aimed to limit the resource-intensive processes of ribosome biogenesis and protect cells from energy deprivation. Stress-induced rDNA repression can be initiated by various mechanisms at different levels. For instance, elevated temperature induces phosphorylation of transcription factor TIF-IA, making it inactive and blocking rDNA transcription initiation [60]. Another example is the eNoSC (energy-dependent nucleolar silencing complex) that enhances rDNA silencing during cellular starvation by histone deacetylation and H3K9 dimethylation at the rDNA promoter [61]. Repression of rRNA synthesis can also be facilitated by stress-dependent inhibition of deacetylation of Pol I subunit PAF53 [62]. Finally, the growing body of evidence indicates that lncRNAs play an important role in the initiation of stress-induced rDNA silencing (Table 1), which will be discussed further. 

## 3. pRNA and PAPAS Induce Repression of rDNA Transcription under Stress

**Figure 2:**
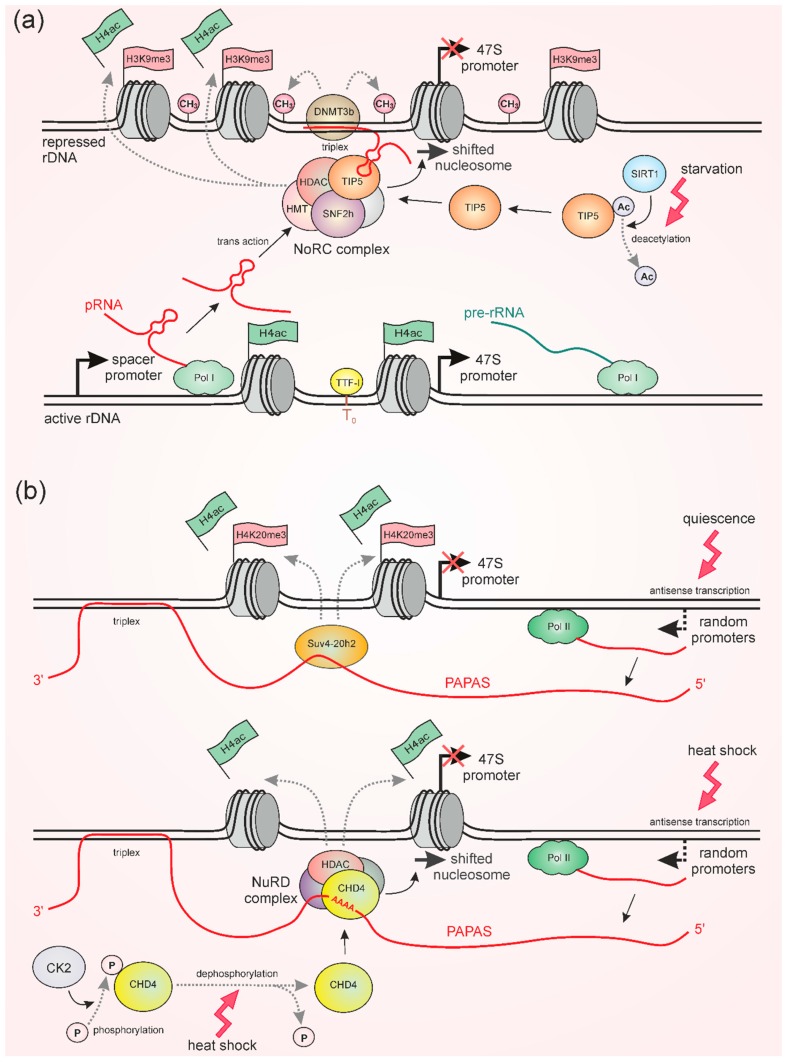
Mechanisms of pRNA and PAPAS action. (**a**) pRNA is transcribed by Pol I from spacer promoters juxtaposed to active rRNA genes. A hairpin structure of pRNA is recognized by TIP5 protein that allows it to form the Nucleolar Remodeling Complex (NoRC). Glucose starvation increases the activity of SIRT1, which deacetylates TIP5 and thereby enhances its association with pRNA. NoRC shifts a promoter nucleosome into a repressive position to block the 47S promoter and provides chromatin repression by histone deacetylation (removal of H4ac marks) and establishment of H3K9me3 modification. The 5′-end of pRNA forms RNA-DNA triplex with T_o_ site of the rDNA promoter. The triplex impairs TTF-I binding and recruits DNMT3b, which methylates DNA (indicated as CH3). (**b**) Quiescence and heat shock activate Pol II-mediated transcription of PAPAS antisense RNAs, which starts from random sites within the rDNA locus. PAPAS is able to anchor by forming DNA:RNA triplex with the enhancer element region in the IGS. Upon quiescence, PAPAS recruits Suv4-20h2, which establishes the repressive H4K20me3 mark at the rDNA. Under heat shock, PAPAS interacts with dephosphorylated CHD4 protein, a component of the NuRD complex that represses rDNA transcription by histone deacetylation and promoter nucleosome shifting.

In studies on murine and human cell lines, the Ingrid Grummt group has found two types of non-coding RNAs which mediate repression of rDNA synthesis: pRNA (promoter associated RNAs) and PAPAS (promoter and pre-rRNA antisense). pRNAs are 150∼300 nt long molecules produced in the same direction as rRNA by RNA Pol I from spacer promoters, which in mice are located ~2 kb upstream of the pre-rRNA transcription start site [63,64] (Figure 2a). In contrast, PAPAS represent a population of antisense RNAs that are synthesized by Pol II. PAPAS transcripts do not have a common promoter, their transcription starts from random sites and can span both pre-rRNA coding and IGS regions being over 10 kb long [67,68] (Figure 2b). Although pRNA and PAPAS interact with distinct proteins and become active upon different conditions, the mechanisms of their action display some common traits. Both RNAs directly interact with rDNA sequences and recruit nucleosome remodeling complexes, as well as special factors producing repressive histone modifications at rDNA promoters.

pRNAs can associate with 47S pre-rRNA promoters forming RNA-DNA triplex structures by Hoogsteen base pairing between DNA and single-stranded RNA [66]. At the same time, pRNA binds the Nucleolar Remodeling Complex (NoRC) and thereby recruits it to the rDNA promoter [63,64] (Figure 2a). The NoRC comprises the SNF2h remodeling protein of the ISWI family and a large subunit, termed TIP5 (TTF1 interacting protein 5) [79]. A specific hairpin structure in pRNA is directly recognized by TIP5 that leads to changes of TIP5 conformation and allows it to associate with other proteins required for NoRC function [64]. Mutations that disrupt the pRNA hairpin structure impede the binding of pRNA by TIP5 and abolish the nucleolar localization of NoRC components [64]. Moreover, pRNAs themselves are thought to be stabilized via TIP5 binding, since TIP5 overexpression increases the amount of pRNAs, whereas TIP5 depletion conversely decreases their level [63]. Functional NoRC, anchored by pRNA to the rDNA promoter, shifts the promoter nucleosome into a repressive position at the transcription start site thereby establishing rDNA silencing by preventing transcription initiation [59]. Furthermore, pRNA-bound TIP5 interacts with histone deacetylases (HDAC), which remove histone acetylation and histone methyltransferases (HMT), which establish the repressive transcriptional marks H3K9me2/3 and H4K20me3 [36,63,80]. In addition, pRNA mediates the association of NoRC with PARP (poly(ADP-ribose)-polymerase-1), ensuring the maintenance of silent rDNA chromatin during cell division by introducing poly(ADP-ribose) histone marks [81].

Under normal conditions, the pRNA-NoRC pathway is required for the silencing of a large fraction of rDNA repeats, which usually exist in a repressive state [63,64,80]. pRNA was shown to be transcribed from spacer promoters of active rRNA genes in the early S phase of the cell cycle and affect transcription of other rDNA repeats in trans during late S phase [80] (Figure 2a). As mentioned above, this repression is necessary to control the level of ribosome synthesis and to ensure the stability of rDNA loci in general. On the other hand, pRNA plays an important role in the cellular response to starvation via more extensive repression of rDNA transcription [65]. This mechanism is based on the (de)acetylation of TIP5, which in turn depends on the energetic status of the cell. Normally, TIP5 interaction with pRNA is regulated by the balanced activities of MOF acetylase and SIRT1 deacetylase. Acetylation of TIP5 by MOF impedes the RNA-binding activity of TIP5, whereas its deacetylation by SIRT1 has the opposite effect, enhancing its association with pRNA [65]. Caloric restriction, e.g., glucose starvation, increases the activity of SIRT1 [82] and facilitates the binding of TIP5 to pRNA [65]. Therefore, starvation strengthens NoRC recruitment to rDNA promoters, which increases heterochromatic histone marks, causes a shift of the promoter-bound nucleosome, and thereby represses rDNA transcription [65]. Thus, stressful conditions might lead to large-scale lncRNA-based repression of rDNA genes due to the post-translational modification of a component of the chromatin remodeling complex. 

Importantly, pRNA is able also to induce rDNA silencing by another mechanism that implicates the aforementioned triplex structure (Figure 2a). Formation of a pRNA–DNA triplex prevents binding of the transcription factor TTF-I to its T_0_ site within the promoter region, initiating transcriptional repression [66]. Moreover, the triplex specifically recruits the DNMT3b DNA methylase, which methylates the rDNA promoter and enhances the repressive effect [66,83]. Notably, it was found that even methylation of a single CpG site in the rDNA promoter can be critical, because it impairs binding of the UBF transcription factor [84]. Although DNA methylation does not depend on the enzymatic activity of the NoRC complex [66], these two methods of silencing may be interrelated. It was shown that upon glucose starvation, pRNA also induces rDNA methylation [65], possibly because deacetylated TIP5 facilitates pRNA-DNA triplex formation via stabilization of pRNA. 

Interestingly, recent data shows that PAPAS lncRNA also forms an RNA-DNA triplex. The 3′ poly(A)-tails of these transcripts interact with poly(T)-sequence (T stretch) at rRNA gene enhancers, which are located at the position −275/−336 upstream of the rDNA transcription start site [69]. Apparently, triplex formation allows for anchoring these lncRNAs near rRNA genes and prohibiting its stochastic spreading, but DNA methylation in the case of PAPAS was not observed. In contrast to pRNA, which is active under normal conditions and during starvation [63,64,65,80], PAPAS induces rDNA repression upon heat shock or hypo-osmotic stresses as well as during cellular quiescence [67,70,71], a state that is characterized by the absence of cell divisions, while the ability to re-enter proliferation is retained. In all cases, PAPAS becomes upregulated, while the mechanisms of PAPAS-mediated rDNA silencing upon quiescence and upon stresses have been shown to be different [67,70,71] (Figure 2b). RNA immunoprecipitation experiments revealed that during cellular quiescence in density-arrested or serum-deprived mouse fibroblasts, elevated levels of PAPAS recruit histone methyltransferase Suv4-20h2, which establishes a repressive H4K20me3 mark at rDNA, whereas H3K9me3 or H3K27me3 abundances are not increased [67]. The heat and hypo-osmotic shocks cause ubiquitin-mediated proteasomal degradation of Suv4-20h2. Nevertheless, in these cases, upregulated PAPAS provides rDNA repression by recruiting the NuRD (nucleosome remodeling and deacetylase) complex [70,71]. Recent work suggests that at elevated temperatures, the NuRD component ATPase CHD4 binds an unstructured A-rich region of PAPAS [69]. Being recruited to the rDNA promoter by PAPAS, the multicomponent NuRD complex performs histone deacetylation and shifts a promoter-bound nucleosome that turns off rDNA transcription (Figure 2b) [70,71] in a similar fashion to the pRNA-NoRC-mediated effect. Intriguingly, CHD4 during heat shock also binds pRNA, albeit less efficiently than PAPAS [69]. Thus, it is plausible that both PAPAS and pRNA can recruit the NuRD complex to rDNA. 

Upon heat stress, PAPAS interaction with CHD4 requires not only an increased number of PAPAS transcripts, but also heat-induced dephosphorylation of CHD4 [69]. Zhao et al. found that at normal temperature, PAPAS fails to interact with CHD4, because it is phosphorylated by CK2 kinase, whereas upon heat shock CK2 is inactivated (Figure 2b). Thus, the choice between the two mechanisms of PAPAS-mediated rDNA repression is determined by the presence of histone methyltransferase Suv4-20h2 upon quiescence or dephosphorylated CHD4 upon heat shock. It is also of interest to find out the mechanisms that define the increased expression of PAPAS in both cases. 

## 4. snoRNA-Containing lncRNAs LoNA and SLERT Regulate rDNA Transcription Through Interaction with Nucleolar Proteins

Small nucleolar RNA (snoRNAs) play an important role in rRNA biogenesis guiding pseudouridylation and fibrillarin dependent 2′-*O*-ribose-methylation [85]. Recently, it was found that some lncRNAs comprising snoRNA sequences are engaged in the modulation of rRNA biogenesis. In contrast to pRNA and PAPAS, these transcripts are produced outside the nucleolus. One of them, called LoNA, was detected in the nucleoli of murine neuroblastoma cell line and in mouse brain [76]. This spliced and polyadenylated 1.5 kb long RNA represses rDNA transcription, causing a decrease of the H3K4me3 mark, an increase of repressive H3K9me2, H3K27me3 marks and preventing UBF loading to rDNA. These effects occur due to the interaction between the 5′-region of LoNA and nucleolin, a known abundant nucleolar multitasking protein involved in rDNA transcription regulation and other steps of ribosome biogenesis ([86,87] for reviews). At the same time, the snoRNA-containing 3′-region of LoNA binds fibrillarin compromising rRNA 2′-*O*-methylation as a competitor with U3 snoRNA in the process of rRNA maturation. Thus, LoNA is suggested to be a regulator of both rDNA transcription and rRNA methylation. It is conceivable also, that LoNA interaction with fibrillarin and nucleolin can affect LLPS-based nucleolar structure. 

A decrease of LoNA level that leads to elevated ribosome production has been shown to correlate with enhanced neuronal activity in the mouse hippocampus [76]. The neurons of patients with Alzheimer disease exhibit decreased rRNA production [88], which was also observed in a mouse model of Alzheimer disease [76]. Interestingly, in the brain of these mice LoNA was upregulated, whereas its knockdown improved learning and memory [76]. It is tempting to suggest also that LoNA is a potential participant in the nucleolar stress response, since Alzheimer disease is known to be accompanied by oxidative stress [89] and nucleolin is stress-responsive protein that controls stability or translation of various mRNAs [86,90].

Another nucleolar snoRNA-containing transcript called SLERT (snoRNA-ended lncRNA enhances pre-ribosomal RNA transcription) was detected in human embryonic stem cells and cancer cells [77]. Contrary to the above-described repressive lncRNAs, *SLERT* emerges as a factor promoting rDNA transcription. While in response to environmental stresses the cell suppresses rRNA synthesis, the opposite situation is observed in cancer cells, which seek to maximize the production of ribosomes [6]. The 694 nt SLERT originates from the intron of a protein-coding gene as a result of unusual alternative splicing [77]. Both ends of SLERT contain snoRNAs necessary for its translocation to the nucleolus. A region adjacent to the 3′-snoRNA sequence of SLERT was shown to be responsible for its association with RNA helicase DDX21, a known participant of ribosome biogenesis. Interestingly, application of SIM (super-resolution structured illumination microscopy) revealed the ring-shaped DDX21 arrangement surrounding multiple Pol I complexes. These DDX21 rings repress rDNA transcription, whereas SLERT binding to these structures prevents repression. At the molecular level, *SLERT* acts as an allosteric repressor inducing “closed” conformation of the DDX21 ATP-binding domain. This conformational change impairs DDX21 interaction with Pol I and thereby allows Pol I-mediated transcription. Contrarily, “open” DDX21 conformation due to *SLERT* depletion causes an increase of DDX21-Pol I association coupled with diminishing of Pol I transcription that leads to reduced proliferation of cancer cells [77]. 

## 5. IGS Transcripts are Involved in the Formation of Stress-Induced Nucleolar Compartments 

A lot of proteins that have no known roles in ribosome biosynthesis are sequestered or released by the nucleolus in response to changes in cellular growth conditions or different stresses [91,92,93]. For some of these proteins, the transient nucleolar localization was shown to be important for temporary inactivation of proteins [74,94,95]. Interestingly, one of many examples of such a function comes from the above-mentioned work concerning PAPAS-mediated heat-dependent DNA repression [69]. The α-subunit of protein kinase CK2 that maintains the CHD4 phosphorylation, thereby preventing rDNA silencing by PAPAS (Figure 2B), was found to be sequestrated into the granular nucleolar compartment upon heat shock [96]. The molecular mechanisms that determine the dynamic nucleolar localization of nucleoplasmic proteins remain incompletely understood, although it is now clear that, at least in some cases, this stress-induced sequestration in the nucleolus depends on special lncRNAs, produced from IGS regions [72,73,74,75]. 

**Figure 3:**
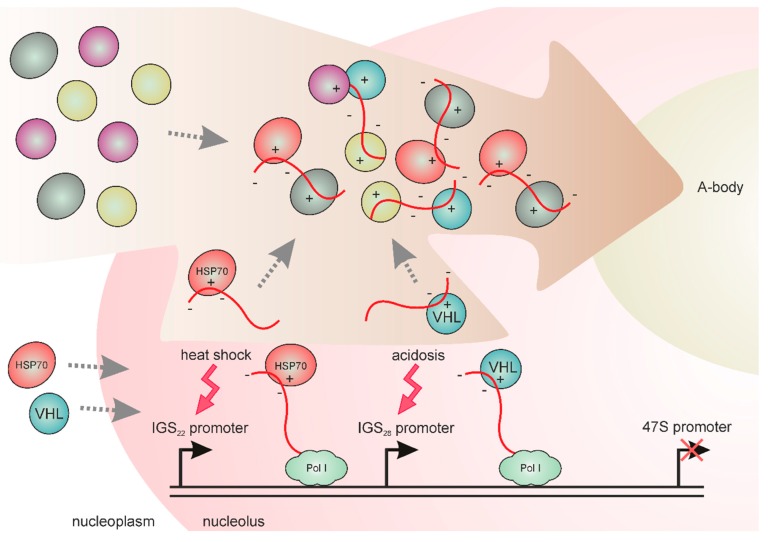
IGSRNAs and A-body formation in human cells. IGS_22_RNA and IGS_28_RNA are transcribed by Pol I from two distinct IGS promoters in response to heat shock and acidosis stress, respectively. These transcripts capture HSP70, VHL and many other nucleoplasmic proteins for their detention in the nucleolus. The electrostatic interactions between cationic amyloid-converting motifs of proteins and negatively charged unstructured IGSRNAs trigger formation of the solid A-body in the nucleolus.

Extended (up to 30 kb in mammals) IGSs comprise promoters of the internal spacer transcripts, which can be separated by many kb from 47S promoters [31]. The group of Stephen Lee has carried out attentive research of the IGS transcripts involved in the stress response in human cell lines [72,73,74,75]. First, it was found that cellular stress during acidosis caused the elevated Pol I-dependent production of the IGS transcripts, which were named IGS_28_RNAs, due to their origin from a discrete IGS region located 28 kb downstream of the pre-rRNA transcription start site [72]. These transcripts are processed into ~300 nt lncRNAs, which accumulate in the nucleolus and associate with several nucleoplasmic proteins, including the ubiquitin ligase VHL [72] (Figure 3). This nucleolar capture inactivates VHL, which normally mediates degradation of the transcription factor HIF which is involved in stress adaptation [97]. The RNAi depletion of IGS transcripts compromises the detention and immobilization of VHL and other proteins to the nucleolus during acidosis [72]. The authors also found that heat shock induces expression of two other specific IGS transcripts, IGS_16_RNA and IGS_22_RNA. Thus, different IGS promoters corresponded to different stress types. Heat shock-inducible transcripts were also able to implement protein detention to the nucleolus, but the set of detained proteins differed from the acidosis-induced set. It was concluded that the IGS comprises different transcriptional units operating independently from each other to recruit nucleoplasmic proteins to the nucleolus in response to environmental cues [72].

Later, Lee and colleagues noted that the stress-induced IGS transcripts are enriched by simple dinucleotide (CU)n/(AG)n repeats lacking predicted folded regions [75]. The RNAs comprising this type of low-complexity repeat are known to participate in the organization of membrane-less cellular condensates. RNA-FISH analysis with IGS probes revealed distinct nucleolar foci, each of which was specific for only one of the stress types, heat shock or acidosis [74]. These transient foci contained mobile proteins forming liquid-like droplets, judging by the results of the fluorescence recovery after photobleaching (FRAP) experiments. With time, a droplet fusion led to the formation of non-dynamic “solid” foci named A-bodies (Amyloid bodies). They are characterized by an amyloid structure [74], a form of protein aggregation into long fibers, observed both in pathological and normal conditions in some cell types [98]. An A-body can accumulate more than 180 proteins, including different functional classes with a predominance of RNA processing and transcription factors important for cell cycle progression [74]. The authors revealed that the preferred protein partner for forming an A-body comprises a short cationic peptide domain named amyloid-converting motif (ACM), as well as fibrillation propensity domain. The electrostatic interactions between cationic ACM and negatively charged low-complexity IGSRNAs were proposed (Figure 3) [74,75].

Thus, IGS transcripts drive protein component concentration, ensuring the start of an amyloidogenic-like program. The meaning of such responses to stress under acidic or high-temperature conditions appears to be the preservation of a significant amount of non-degraded proteins in a dormant state [74,75]. Moreover, the nucleolar sequestration of proteins plays an important role in the impoverishment of the interactome of the nucleoplasm inducing the global rearrangement of regulatory networks in a stress-survival mode. In addition, transformation of the nucleolus into the solid-like stress compartment may be one of the factors that determine rDNA transcription inactivation [73]. Interestingly, A-bodies can be disaggregated by Hsp chaperones, demonstrating the reversibility of the observed “physiological amyloidogenesis” [74]. 

The understanding of the regulation of IGSRNA transcription remains poor. Since different IGSRNAs emerge in human rDNA spacers upon different stresses [72,74], activation of their transcription seems to be a quite complex process. Moreover, it is not clear how many different transcription units are lurking in IGS and why the cell requires such a large set of stimuli specific IGSRNAs. The complexity of various IGS regions regulation is further exemplified by the finding of other IGSRNA variants (IGS_36_RNA and IGS_39_RNA), which are specifically activated under certain conditions accompanied by the inhibition of rRNA synthesis [99].

Given that a clear function of IGSRNAs in the process of protein sequestration in the nucleolus has so far only been shown in certain human cell cultures [72,73,74,75], it is of interest to find out how widespread and conservative the IGSRNA-based mechanism is. In our work, we have found that in *Drosophila melanogaster,* heat shock caused a drastic redistribution of the nucleoplasmic protein Piwi to the nucleolus [100]. Importantly, this nucleolar detention of Piwi occurred only in the presence of Pol I-mediated transcription, even though rRNA synthesis was repressed under heat shock. These results imply that specific stress-induced Pol I transcripts may mediate nucleolar sequestration of proteins in *D. melanogaster*. 

In addition to the role of IGS transcripts in titrating important nucleoplasmic proteins, their involvement in the formation of the perinuclear compartment (РNC) was recently revealed [78]. The PNC body, the functions of which are obscure, is found mainly in cancer cells and accumulates Pol III transcription products [101]. In HeLa cells, the PNC was shown to contain Pol I-produced ~10 kb-long IGS noncoding transcripts called PNCTRs [78], which include a region identical to IGS_28_RNA described earlier as acidosis-inducible transcript [72]. PNCTRs also comprise motifs capable of recruiting multiple copies of the pre-mRNA processing factor PTBP1/hnRNP I (polypyrimidine tract binding protein), known in particular as a repressor of differentiation-specific alternative splicing [78]. PNCTR depletion causes a strong reduction of the PNC body and triggers apoptosis, pointing at its potential pro-survival function [78]. 

Thus, IGS transcripts can have a dual function, providing both the nucleolar response to stressful situations as well as determining PNC body formation in cancer cells.

## 6. Concluding Remarks 

The data discussed here demonstrate the diversity of nucleolar lncRNAs, which originate both from the nucleolar organizer itself (pRNA, PAPAS, IGSRNA, PNCTR) and from remote regions of the genome outside the rDNA cluster (LoNA, SLERT). Importantly, most of these RNAs are present at low level or nearly absent in cells under normal conditions. Accumulation of nucleolar lncRNAs is observed in response to different stresses (upregulation of IGSRNA and PAPAS) and unusual cellular states like quiescence (induction of PAPAS) or tumorigenesis (accumulation of SLERT and PNCTR) (Table 1). However, the mechanisms, which provide stimulus-specific activation of these transcripts, remain unknown in most cases. One of the quite clear ways of stress-dependent accumulation of lncRNA is represented by the stabilization of pRNA, which occurs under glucose deprivation due to the facilitated interaction with TIP5 protein [65]. It is also noteworthy that the nucleolar response to stress through pRNA and PAPAS can be initiated by specific post-translational modifications of their protein partners (deacetylation and dephosphorylation, respectively), which in turn triggers lncRNA-dependent repression of rDNA [65,69]. This principle may be beneficial due to its high speed, since it does not require additional protein synthesis.

Several basic types of nucleolar lncRNA molecular functions can be distinguished. The first role is exemplified by pRNA and PAPAS, which can interact with rDNA by RNA:DNA triplex formation and recruit protein complexes regulating chromatin state and transcription [63,64,66,67,68,69,70,71]. Secondly, lncRNAs can specifically bind certain nucleolar proteins, causing their inactivation or distracting them from the activities associated with rRNA biogenesis. These functions can be attributed to LoNA, which interacts with nucleolin and fibrillarin [76], and to SLERT, which acts as an allosteric effector changing the conformation of DDX21 RNA-helicase [77]. Notably, LoNA suppresses rRNA synthesis, whereas SLERT enhances it. Another example of this functional type is human lncRNA, called circANRIL, which binds the rRNA maturation factor PES1, thereby impairing ribosome biogenesis and preventing atherosclerosis by inducing apoptosis in a fraction of highly proliferative vascular cells [102]. Thirdly, lncRNAs carrying simple repeats are involved in sequestration of proteins and formation of specific subcompartments. Upon stress, IGSRNAs can form A-bodies inside the nucleolus, triggering the process of transition of the liquid phase to a solid state [74,75]. PNCTR transcripts, also produced from IGS regions, participate in the assembly of the perinucleolar compartment, a functional role for which remains unknown [78]. Interestingly, another type of non-coding RNAs (aluRNAs) are important for maintaining the structure of the nucleolus itself [26], although the molecular principles of this function are not fully understood. Beyond the nucleolus, the involvement of lncRNAs in the formation of nuclear bodies has been demonstrated for paraspeckles [103,104], as well as for ω-speckles found in nuclei of *Drosophila melanogaster* [105]. 

Our knowledge of the various mechanisms of nucleolus-associated lncRNA action is constantly increasing and is not limited to the cases described in this review. For example, a special biological role is performed by the X-linked lncRNA Firre, which is required to anchor the inactive X chromosome in female cells to the nucleolus surface [106]. Recently it was shown that antisense lncRNA EPB41L4A-AS1 in human cancer cells is responsible for the translocation of histone-deacetylase HDAC2 to the nucleolus due to the interaction with both HDAC2 and nucleophosmin (NPM1) [107]. Given that both NoRC and NuRD repressive complexes include HDAC1/2 as necessary components [83,108], the shuttling of HDAC2 between the nucleoplasm and the nucleolus may potentially be required for functioning of these complexes in rDNA repression.

Altogether, the discussed studies performed mainly on human and murine cell cultures demonstrate an extreme variety of functions of lncRNAs associated with the nucleolus. In the future, it would be interesting to investigate similar mechanisms at the organism level, as well as exploring the evolutionary aspect, extending the nucleolar lncRNA research to other model organisms.

## Figures and Tables

**Table 1:** lncRNAs in the nucleoli under stresses and different cell states.

lncRNA	Partner Proteins	Conditions	Function	Length, nt	References
pRNA	NoRC (TIP5) DNMT3b	Normal state Starvation	rDNA repression	150–300	[63,64,65,66]
PAPAS	NuRD (CHD4) Suv4-20h2	Quiescence Heat shock Hypo-osmotic shock	rDNA repression	>10,000	[67,68,69,70,71]
IGS_22_RNA IGS_16_RNA	HSP70 and other	Heat shock	Protein sequestration	~300	[72,73,74,75]
IGS_28_RNA	VHL and other	Acidosis	Protein sequestration	~300	[72,73,74,75]
LoNA	nucleolin fibrillarin	?	rDNA repression	~1500	[76]
SLERT	DDX21	Carcinogenesis	rDNA activation	694	[77]
PNCTR	PTBP1	Carcinogenesis	Protein sequestration PNC formation	>10,000	[78]

## References

[1] Yao R., Wang Y., Chen L. (2019). Cellular functions of long noncoding RNAs. Nat. Cell Biol..

[2] Rinn J.L., Chang H.Y. (2012). Genome regulation by long noncoding RNAs. Annu. Rev. Biochem..

[3] Boyd M.T., Vlatkovic N., Rubbi C.P. (2011). The nucleolus directly regulates p53 export and degradation. J. Cell Biol..

[4] Lindström M.S., Jurada D., Bursac S., Orsolic I., Bartek J., Volarevic S. (2018). Nucleolus as an emerging hub in maintenance of genome stability and cancer pathogenesis. Oncogene.

[5] Lam Y.W., Trinkle-Mulcahy L. (2015). New insights into nucleolar structure and function. F1000Prime Rep..

[6] Nguyen L.X.T., Raval A., Garcia J.S., Mitchell B.S. (2015). Regulation of Ribosomal Gene Expression in Cancer. J. Cell. Physiol..

[7] Núñez Villacís L., Wong M.S., Ferguson L.L., Hein N., George A.J., Hannan K.M. (2018). New Roles for the Nucleolus in Health and Disease. BioEssays.

[8] Fulda S., Gorman A.M., Hori O., Samali A. (2010). Cellular stress responses: Cell survival and cell death. Int. J. Cell Biol..

[9] Hartl F.U. (2017). Protein Misfolding Diseases. Annu. Rev. Biochem..

[10] Pilla E., Schneider K., Bertolotti A. (2017). Coping with Protein Quality Control Failure. Annu. Rev. Cell Dev. Biol..

[11] Grummt I. (2013). The nucleolus—guardian of cellular homeostasis and genome integrity. Chromosoma.

[12] Valadkhan S., Valencia-Hipólito A. (2015). lncRNAs in Stress Response. Current Topics in Microbiology and Immunology.

[13] Lakhotia S.C. (2012). Long non-coding RNAs coordinate cellular responses to stress. Wiley Interdiscip. Rev. RNA.

[14] Verheyden Y., Goedert L., Leucci E. (2019). Control of nucleolar stress and translational reprogramming by lncRNAs. Cell Stress.

[15] Hyman A.A., Weber C.A., Jülicher F. (2014). Liquid-Liquid Phase Separation in Biology. Annu. Rev. Cell Dev. Biol..

[16] Alberti S., Gladfelter A., Mittag T. (2019). Considerations and Challenges in Studying Liquid-Liquid Phase Separation and Biomolecular Condensates. Cell.

[17] Shin Y., Brangwynne C.P. (2017). Liquid phase condensation in cell physiology and disease. Science.

[18] Sawyer I.A., Sturgill D., Dundr M. (2019). Membraneless nuclear organelles and the search for phases within phases. Wiley Interdiscip. Rev. RNA.

[19] Banani S.F., Lee H.O., Hyman A.A., Rosen M.K. (2017). Biomolecular condensates: Organizers of cellular biochemistry. Nat. Rev. Mol. Cell Biol..

[20] Van Treeck B., Protter D.S.W., Matheny T., Khong A., Link C.D., Parker R. (2018). RNA self-assembly contributes to stress granule formation and defining the stress granule transcriptome. Proc. Natl. Acad. Sci. USA.

[21] Fay M.M., Anderson P.J. (2018). The Role of RNA in Biological Phase Separations. J. Mol. Biol..

[22] Drino A., Schaefer M.R. (2018). RNAs, Phase Separation, and Membrane-Less Organelles: Are Post-Transcriptional Modifications Modulating Organelle Dynamics?. BioEssays.

[23] Jain A., Vale R.D. (2017). RNA phase transitions in repeat expansion disorders. Nature.

[24] Mitrea D.M., Cika J.A., Guy C.S., Ban D., Banerjee P.R., Stanley C.B., Nourse A., Deniz A.A., Kriwacki R.W. (2016). Nucleophosmin integrates within the nucleolus via multi-modal interactions with proteins displaying R-rich linear motifs and rRNA. Elife.

[25] Feric M., Vaidya N., Harmon T.S., Mitrea D.M., Zhu L., Richardson T.M., Kriwacki R.W., Pappu R.V., Brangwynne C.P. (2016). Coexisting Liquid Phases Underlie Nucleolar Subcompartments. Cell.

[26] Caudron-Herger M., Pankert T., Seiler J., Németh A., Voit R., Grummt I., Rippe K. (2015). Alu element-containing RNAs maintain nucleolar structure and function. EMBO J..

[27] Caudron-Herger M., Pankert T., Rippe K. (2016). Regulation of nucleolus assembly by non-coding RNA polymerase II transcripts. Nucleus..

[28] McStay B. (2016). Nucleolar organizer regions: Genomic “dark matter” requiring illumination. Genes Dev..

[29] De Winter R.F., Moss T. (1986). Spacer promoters are essential for efficient enhancement of X. laevis ribosomal transcription. Cell.

[30] Kuhn A., Grummt I. (1987). A novel promoter in the mouse rDNA spacer is active in vivo and in vitro. EMBO J..

[31] Grimaldi G., Di Nocera P.P. (1988). Multiple repeated units in Drosophila melanogaster ribosomal DNA spacer stimulate rRNA precursor transcription. Proc. Natl. Acad. Sci. USA.

[32] Stefanovsky V.Y., Pelletier G., Hannan R., Gagnon-Kugler T., Rothblum L.I., Moss T. (2001). An immediate response of ribosomal transcription to growth factor stimulation in mammals is mediated by ERK phosphorylation of UBF. Mol. Cell.

[33] Drakas R., Tu X., Baserga R. (2004). Control of cell size through phosphorylation of upstream binding factor 1 by nuclear phosphatidylinositol 3-kinase. Proc. Natl. Acad. Sci. USA.

[34] Voit R., Hoffmann M., Grummt I. (1999). Phosphorylation by G1-specific cdk-cyclin complexes activates the nucleolar transcription factor UBF. EMBO J..

[35] Peng J.C., Karpen G.H. (2007). H3K9 methylation and RNA interference regulate nucleolar organization and repeated DNA stability. Nat. Cell Biol..

[36] Guetg C., Lienemann P., Sirri V., Grummt I., Hernandez-Verdun D., Hottiger M.O., Fussenegger M., Santoro R. (2010). The NoRC complex mediates the heterochromatin formation and stability of silent rRNA genes and centromeric repeats. EMBO J..

[37] Conconi A., Widmer R.M., Koller T., Sogo J.M. (1989). Two different chromatin structures coexist in ribosomal RNA genes throughout the cell cycle. Cell.

[38] Stancheva I., Lucchini R., Koller T., Sogo J.M. (1997). Chromatin structure and methylation of rat rRNA genes studied by formaldehyde fixation and psoralen cross-linking. Nucleic Acids Res..

[39] Roussel P., André C., Comai L., Hernandez-Verdun D. (1996). The rDNA transcription machinery is assembled during mitosis in active NORs and absent in inactive NORs. J. Cell Biol..

[40] Zillner K., Komatsu J., Filarsky K., Kalepu R., Bensimon A., Németh A. (2015). Active human nucleolar organizer regions are interspersed with inactive rDNA repeats in normal and tumor cells. Epigenomics.

[41] Caburet S., Conti C., Schurra C., Lebofsky R., Edelstein S.J., Bensimon A. (2005). Human ribosomal RNA gene arrays display a broad range of palindromic structures. Genome Res..

[42] Eickbush T.H., Eickbush D.G. (2015). Integration, Regulation, and Long-Term Stability of R2 Retrotransposons. Microbiol Spectr..

[43] Moss T., Mars J.-C., Tremblay M.G., Sabourin-Felix M. (2019). The chromatin landscape of the ribosomal RNA genes in mouse and human. Chromosom. Res..

[44] Goodfellow S.J., Zomerdijk J.C.B.M. (2013). Basic mechanisms in RNA polymerase I transcription of the ribosomal RNA genes. Subcell. Biochem..

[45] Hamdane N., Stefanovsky V.Y., Tremblay M.G., Németh A., Paquet E., Lessard F., Sanij E., Hannan R., Moss T. (2014). Conditional Inactivation of Upstream Binding Factor Reveals Its Epigenetic Functions and the Existence of a Somatic Nucleolar Precursor Body. PLoS Genet..

[46] Herdman C., Mars J.-C., Stefanovsky V.Y., Tremblay M.G., Sabourin-Felix M., Lindsay H., Robinson M.D., Moss T. (2017). A unique enhancer boundary complex on the mouse ribosomal RNA genes persists after loss of Rrn3 or UBF and the inactivation of RNA polymerase I transcription. PLOS Genet..

[47] Mars J.-C., Sabourin-Felix M., Tremblay M.G., Moss T. (2018). A Deconvolution Protocol for ChIP-Seq Reveals Analogous Enhancer Structures on the Mouse and Human Ribosomal RNA Genes. G3: Genes|Genomes|Genetics.

[48] Zentner G.E., Saiakhova A., Manaenkov P., Adams M.D., Scacheri P.C. (2011). Integrative genomic analysis of human ribosomal DNA. Nucleic Acids Res..

[49] Diermeier S.D., Németh A., Rehli M., Grummt I., Längst G. (2013). Chromatin-Specific Regulation of Mammalian rDNA Transcription by Clustered TTF-I Binding Sites. PLoS Genet..

[50] Längst G., Becker P.B., Grummt I. (1998). TTF-I determines the chromatin architecture of the active rDNA promoter. EMBO J..

[51] Santoro R., Li J., Grummt I. (2002). The nucleolar remodeling complex NoRC mediates heterochromatin formation and silencing of ribosomal gene transcription. Nat. Genet..

[52] Németh A., Guibert S., Tiwari V.K., Ohlsson R., Längst G. (2008). Epigenetic regulation of TTF-I-mediated promoter–terminator interactions of rRNA genes. EMBO J..

[53] Denissov S., Lessard F., Mayer C., Stefanovsky V., van Driel M., Grummt I., Moss T., Stunnenberg H.G. (2011). A model for the topology of active ribosomal RNA genes. EMBO Rep..

[54] Potapova T.A., Gerton J.L. (2019). Ribosomal DNA and the nucleolus in the context of genome organization. Chromosom. Res..

[55] Schöfer C., Weipoltshammer K. (2018). Nucleolus and chromatin. Histochem. Cell Biol..

[56] Hamperl S., Wittner M., Babl V., Perez-Fernandez J., Tschochner H., Griesenbeck J. (2013). Chromatin states at ribosomal DNA loci. Biochim. Biophys. Acta—Gene Regul. Mech..

[57] Srivastava R., Srivastava R., Ahn S.H. (2016). The Epigenetic Pathways to Ribosomal DNA Silencing. Microbiol. Mol. Biol. Rev..

[58] Xie W., Ling T., Zhou Y., Feng W., Zhu Q., Stunnenberg H.G., Grummt I., Tao W. (2012). The chromatin remodeling complex NuRD establishes the poised state of rRNA genes characterized by bivalent histone modifications and altered nucleosome positions. Proc. Natl. Acad. Sci..

[59] Li J., Längst G., Grummt I. (2006). NoRC-dependent nucleosome positioning silences rRNA genes. EMBO J..

[60] Bierhoff H., Dundr M., Michels A.A., Grummt I. (2008). Phosphorylation by Casein Kinase 2 Facilitates rRNA Gene Transcription by Promoting Dissociation of TIF-IA from Elongating RNA Polymerase I. Mol. Cell. Biol..

[61] Murayama A., Ohmori K., Fujimura A., Minami H., Yasuzawa-Tanaka K., Kuroda T., Oie S., Daitoku H., Okuwaki M., Nagata K. (2008). Epigenetic Control of rDNA Loci in Response to Intracellular Energy Status. Cell.

[62] Chen S., Seiler J., Santiago-Reichelt M., Felbel K., Grummt I., Voit R. (2013). Repression of RNA polymerase I upon stress is caused by inhibition of RNA-dependent deacetylation of PAF53 by SIRT7. Mol. Cell.

[63] Mayer C., Schmitz K.-M., Li J., Grummt I., Santoro R. (2006). Intergenic Transcripts Regulate the Epigenetic State of rRNA Genes. Mol. Cell.

[64] Mayer C., Neubert M., Grummt I. (2008). The structure of NoRC-associated RNA is crucial for targeting the chromatin remodelling complex NoRC to the nucleolus. EMBO Rep..

[65] Zhou Y., Schmitz K.-M., Mayer C., Yuan X., Akhtar A., Grummt I. (2009). Reversible acetylation of the chromatin remodelling complex NoRC is required for non-coding RNA-dependent silencing. Nat. Cell Biol..

[66] Schmitz K.-M., Mayer C., Postepska A., Grummt I. (2010). Interaction of noncoding RNA with the rDNA promoter mediates recruitment of DNMT3b and silencing of rRNA genes. Genes Dev..

[67] Bierhoff H., Dammert M.A., Brocks D., Dambacher S., Schotta G., Grummt I. (2014). Quiescence-Induced LncRNAs Trigger H4K20 Trimethylation and Transcriptional Silencing. Mol. Cell.

[68] Bierhoff H., Schmitz K., Maass F., Ye J., Grummt I. (2010). Noncoding Transcripts in Sense and Antisense Orientation Regulate the Epigenetic State of Ribosomal RNA Genes. Cold Spring Harb. Symp. Quant. Biol..

[69] Zhao Z., Sentürk N., Song C., Grummt I. (2018). lncRNA PAPAS tethered to the rDNA enhancer recruits hypophosphorylated CHD4/NuRD to repress rRNA synthesis at elevated temperatures. Genes Dev..

[70] Zhao Z., Dammert M.A., Grummt I., Bierhoff H. (2016). lncRNA-Induced Nucleosome Repositioning Reinforces Transcriptional Repression of rRNA Genes upon Hypotonic Stress. Cell Rep..

[71] Zhao Z., Dammert M.A., Hoppe S., Bierhoff H., Grummt I. (2016). Heat shock represses rRNA synthesis by inactivation of TIF-IA and lncRNA-dependent changes in nucleosome positioning. Nucleic Acids Res..

[72] Audas T.E., Jacob M.D., Lee S. (2012). Immobilization of proteins in the nucleolus by ribosomal intergenic spacer noncoding RNA. Mol. Cell.

[73] Jacob M.D., Audas T.E., Uniacke J., Trinkle-Mulcahy L., Lee S. (2013). Environmental cues induce a long noncoding RNA-dependent remodeling of the nucleolus. Mol. Biol. Cell.

[74] Audas T.E., Audas D.E., Jacob M.D., Ho J.J.D., Khacho M., Wang M., Perera J.K., Gardiner C., Bennett C.A., Head T. (2016). Adaptation to Stressors by Systemic Protein Amyloidogenesis. Dev. Cell.

[75] Wang M., Tao X., Jacob M.D., Bennett C.A., Ho J.J.D., Gonzalgo M.L., Audas T.E., Lee S. (2018). Stress-Induced Low Complexity RNA Activates Physiological Amyloidogenesis. Cell Rep..

[76] Li D., Zhang J., Wang M., Li X., Gong H., Tang H., Chen L., Wan L., Liu Q. (2018). Activity dependent LoNA regulates translation by coordinating rRNA transcription and methylation. Nat. Commun..

[77] Xing Y.-H., Yao R.-W., Zhang Y., Guo C.-J., Jiang S., Xu G., Dong R., Yang L., Chen L.-L. (2017). SLERT Regulates DDX21 Rings Associated with Pol I Transcription. Cell.

[78] Yap K., Mukhina S., Zhang G., Tan J.S.C., Ong H.S., Makeyev E.V. (2018). A Short Tandem Repeat-Enriched RNA Assembles a Nuclear Compartment to Control Alternative Splicing and Promote Cell Survival. Mol. Cell.

[79] Strohner R., Nemeth A., Jansa P., Hofmann-Rohrer U., Santoro R., Längst G., Grummt I. (2001). NoRC—A novel member of mammalian ISWI-containing chromatin remodeling machines. EMBO J..

[80] Santoro R., Schmitz K.-M., Sandoval J., Grummt I. (2010). Intergenic transcripts originating from a subclass of ribosomal DNA repeats silence ribosomal RNA genes in trans. EMBO Rep..

[81] Guetg C., Scheifele F., Rosenthal F., Hottiger M.O., Santoro R. (2012). Inheritance of Silent rDNA Chromatin Is Mediated by PARP1 via Noncoding RNA. Mol. Cell.

[82] Rodgers J.T., Lerin C., Haas W., Gygi S.P., Spiegelman B.M., Puigserver P. (2005). Nutrient control of glucose homeostasis through a complex of PGC-1α and SIRT1. Nature.

[83] Grummt I., Längst G. (2013). Epigenetic control of RNA polymerase I transcription in mammalian cells. Biochim. Biophys. Acta—Gene Regul. Mech..

[84] Santoro R., Grummt I. (2001). Molecular mechanisms mediating methylation-dependent silencing of ribosomal gene transcription. Mol. Cell.

[85] Kiss T. (2002). Small nucleolar RNAs: An abundant group of noncoding RNAs with diverse cellular functions. Cell.

[86] Abdelmohsen K., Gorospe M. (2012). RNA-binding protein nucleolin in disease. RNA Biol..

[87] McStay B., Grummt I. (2008). The Epigenetics of rRNA Genes: From Molecular to Chromosome Biology. Annu. Rev. Cell Dev. Biol..

[88] Dönmez-Altuntaş H., Akalın H., Karaman Y., Demirtaş H., İmamoğlu N., Özkul Y. (2005). Evaluation of the Nucleolar Organizer Regions in Alzheimer’s Disease. Gerontology.

[89] Chen Z., Zhong C. (2014). Oxidative stress in Alzheimer’s disease. Neurosci. Bull..

[90] Zhang X., Xiao S., Rameau R.D., Devany E., Nadeem Z., Caglar E., Ng K., Kleiman F.E., Saxena A. (2018). Nucleolin phosphorylation regulates PARN deadenylase activity during cellular stress response. RNA Biol..

[91] Mekhail K., Khacho M., Carrigan A., Hache R.R.J., Gunaratnam L., Lee S. (2005). Regulation of ubiquitin ligase dynamics by the nucleolus. J. Cell Biol..

[92] Boulon S., Westman B.J., Hutten S., Boisvert F.-M., Lamond A.I. (2010). The nucleolus under stress. Mol. Cell.

[93] Andersen J.S., Lam Y.W., Leung A.K.L., Ong S.-E., Lyon C.E., Lamond A.I., Mann M. (2005). Nucleolar proteome dynamics. Nature.

[94] Emmott E., Hiscox J.A. (2009). Nucleolar targeting: The hub of the matter. EMBO Rep..

[95] Pederson T., Tsai R.Y.L. (2009). In search of nonribosomal nucleolar protein function and regulation. J. Cell Biol..

[96] Gerber D.A., Souquere-Besse S., Puvion F., Dubois M.-F., Bensaude O., Cochet C. (2000). Heat-induced Relocalization of Protein Kinase CK2. J. Biol. Chem..

[97] Haase V.H. (2009). The VHL tumor suppressor: Master regulator of HIF. Curr. Pharm. Des..

[98] Knowles T.P.J., Vendruscolo M., Dobson C.M. (2014). The amyloid state and its association with protein misfolding diseases. Nat. Rev. Mol. Cell Biol..

[99] Todd M.A.M., Huh M.S., Picketts D.J. (2016). The sub-nucleolar localization of PHF6 defines its role in rDNA transcription and early processing events. Eur. J. Hum. Genet..

[100] Mikhaleva E.A., Leinsoo T.A., Ishizu H., Gvozdev V.A., Klenov M.S. (2019). The nucleolar transcriptome regulates Piwi shuttling between the nucleolus and the nucleoplasm. Chromosom. Res..

[101] Norton J.T., Huang S. (2013). The perinucleolar compartment: RNA metabolism and cancer. Cancer Treat. Res..

[102] Holdt L.M., Stahringer A., Sass K., Pichler G., Kulak N.A., Wilfert W., Kohlmaier A., Herbst A., Northoff B.H., Nicolaou A. (2016). Circular non-coding RNA ANRIL modulates ribosomal RNA maturation and atherosclerosis in humans. Nat. Commun..

[103] Clemson C.M., Hutchinson J.N., Sara S.A., Ensminger A.W., Fox A.H., Chess A., Lawrence J.B. (2009). An architectural role for a nuclear noncoding RNA: NEAT1 RNA is essential for the structure of paraspeckles. Mol. Cell.

[104] Sasaki Y.T.F., Ideue T., Sano M., Mituyama T., Hirose T. (2009). MENε/β noncoding RNAs are essential for structural integrity of nuclear paraspeckles. Proc. Natl. Acad. Sci. USA.

[105] Singh A.K., Lakhotia S.C. (2015). Dynamics of hnRNPs and omega speckles in normal and heat shocked live cell nuclei of Drosophila melanogaster. Chromosoma.

[106] Yang F., Deng X., Ma W., Berletch J.B., Rabaia N., Wei G., Moore J.M., Filippova G.N., Xu J., Liu Y. (2015). The lncRNA Firre anchors the inactive X chromosome to the nucleolus by binding CTCF and maintains H3K27me3 methylation. Genome Biol..

[107] Liao M., Liao W., Xu N., Li B., Liu F., Zhang S., Wang Y., Wang S., Zhu Y., Chen D. (2019). LncRNA EPB41L4A-AS1 regulates glycolysis and glutaminolysis by mediating nucleolar translocation of HDAC2. EBioMedicine.

[108] Lai A.Y., Wade P.A. (2011). Cancer biology and NuRD: A multifaceted chromatin remodelling complex. Nat. Rev. Cancer.

